# Synergistic inhibition of the APC/C by the removal of APC15 in HCT116 cells lacking UBE2C

**DOI:** 10.1242/bio.020842

**Published:** 2016-09-02

**Authors:** Dimitriya H. Garvanska, Marie Sofie Yoo Larsen, Jakob Nilsson

**Affiliations:** The Novo Nordisk Foundation Center for Protein Research, Faculty of Health and Medical Sciences, University of Copenhagen, Blegdamsvej 3B, Copenhagen 2200, Denmark

**Keywords:** UBE2C, UBE2S, APC/C, SAC silencing, APC15, MCC

## Abstract

The spindle assembly checkpoint (SAC) inhibits the anaphase-promoting complex/cyclosome (APC/C) in response to unattached kinetochores by generating a diffusible inhibitor termed the mitotic checkpoint complex (MCC). At metaphase, rapid activation of the APC/C requires removal of the MCC, a process that has been shown to depend on the APC/C E2 enzymes, UBE2C and UBE2S. Here we investigate the *in vivo* role of the APC/C E2 enzymes in SAC silencing using CRISPR/Cas9 genetically engineered HCT116 UBE2C or UBE2S null cell lines. Using live cell assays, we show that UBE2C and UBE2S make a minor contribution to SAC silencing in HCT116 cells. Strikingly, in cells specifically lacking UBE2C, we observe a strong synergistic inhibition of mitotic progression when we stabilize the MCC on the APC/C by depleting APC15, potentially reflecting increased competition between the MCC and the remaining initiating E2 enzyme UBE2D. In conclusion, we provide *in vivo* insight into the APC/C E2 module and its interplay with SAC silencing components.

## Abstract (precis)

**Summary:** The activation of the anaphase-promoting complex drives mitotic exit and here mechanistic aspects of this are explored using CRISPR/Cas9 genetically engineered human null cell lines.

## INTRODUCTION

The APC/C is a large ubiquitin ligase regulating mitotic progression by targeting proteins for ubiquitin-mediated destruction (Pines, 2011). An important substrate of the APC/C is cyclin B1 since the destruction of this protein results in entry into anaphase. The destruction of cyclin B1 is fully dependent on the mitotic co-activator Cdc20 that, together with the APC/C, recognizes destruction motifs in cyclin B1 (Pines, 2011). Furthermore, the poly-ubiquitination of cyclin B1 depends on APC/C-specific E2 enzymes and in collaboration with the Choudhary lab, we have recently showed that at least three E2 enzymes can work with the APC/C *in vivo*, namely UBE2C (UBCH10), UBE2S and UBE2D (UBCH5) (Wild et al., 2016). UBE2C and UBE2D work with the APC/C to add the initial ubiquitin molecules to a substrate that is then subsequently extended by UBE2S that catalyzes the formation of Lys11-linked chains on substrates (Garnett et al., 2009; Williamson et al., 2009; Wu et al., 2010). Interestingly, UBE2C (and likely UBE2D) is recruited to the APC/C via a distinct mechanism from UBE2S (Brown et al., 2015, 2014; Chang et al., 2015). UBE2C interacts with the APC11 RING finger and APC2 in the central cavity of the APC/C complex while UBE2S interacts through a unique interaction surface that positions UBE2S on the side of the APC/C (Brown et al., 2016).

During mitosis the APC/C is inhibited by the SAC that in response to improperly attached kinetochores generates a diffusible inhibitor of the APC/C referred to as the mitotic checkpoint complex (MCC) (Lischetti and Nilsson, 2015; Sacristan and Kops, 2015). The MCC is composed of Cdc20 bound to the checkpoint protein Mad2 and the checkpoint complex BubR1-Bub3, and this complex can bind stably to the APC/C complex that is already bound to Cdc20 (Chao et al., 2012; Hein and Nilsson, 2014; Herzog et al., 2009; Izawa and Pines, 2014; Sudakin et al., 2001). The MCC binds to the central cavity of the APC/C and makes contact with the APC11/APC2 module and blocks UBE2C binding (Alfieri et al., 2016; Chang et al., 2014; Herzog et al., 2009; Yamaguchi et al., 2016). When all kinetochores have properly attached, the MCC needs to be disassembled in order to activate APC/C-Cdc20 and this is an active process. Several proteins have been implicated in MCC disassembly: (1) p31^comet^ in concert with TRIP13 that facilitates removal of Mad2 from the MCC and (2) the small APC/C subunit, APC15 (Mnd2 in budding yeast), the removal of which results in increased levels of MCC on the APC/C and slows APC/C activation (Eytan et al., 2014; Foster and Morgan, 2012; Mansfeld et al., 2011; Miniowitz-Shemtov et al., 2015; Teichner et al., 2011; Uzunova et al., 2012; Wang et al., 2014; Westhorpe et al., 2011; Ye et al., 2015). In addition, the ubiquitination of Cdc20 by the APC/C has been proposed to be required for MCC disassembly in a process dependent on UBE2C and UBE2S (Kelly et al., 2014; Reddy et al., 2007; Williamson et al., 2009). However, the function of Cdc20 ubiquitination, and thus UBE2C and UBE2S, in SAC silencing is debated and conflicting results exist in the literature (Foster and Morgan, 2012; Garnett et al., 2009; Mansfeld et al., 2011; Nilsson et al., 2008; Reddy et al., 2007; Uzunova et al., 2012; Varetti et al., 2011).

The role of the E2 enzymes and their interplay with SAC silencing factors is important to clarify because timely activation of the APC/C is critical for accurate chromosome segregation. Here, we analyze SAC silencing *in vivo* using genetically engineered HCT116 E2 null cell lines. We find a minor contribution of the E2 module in SAC silencing in these HCT116 cell lines but we observe a pronounced mitotic delay when we deplete APC15 in HCT116 cells lacking UBE2C. This strong synergistic effect is SAC dependent and we speculate that APC15 removal, through the MCC, interferes with UBE2D function. Our work thus provides *in vivo* insight into the regulation of the APC/C and the role of the E2 enzymes and APC15.

## RESULTS

### Analysis of SAC silencing in HCT116 cells lacking specific APC/C E2 enzymes

We recently reported the generation of genetically engineered HCT116 cell lines where the UBE2C and UBE2S genes were deleted by CRISPR/Cas9 technology, providing an excellent model system for testing the role of these E2 enzymes in SAC silencing *in vivo* (Wild et al., 2016).

**Fig. 1.**
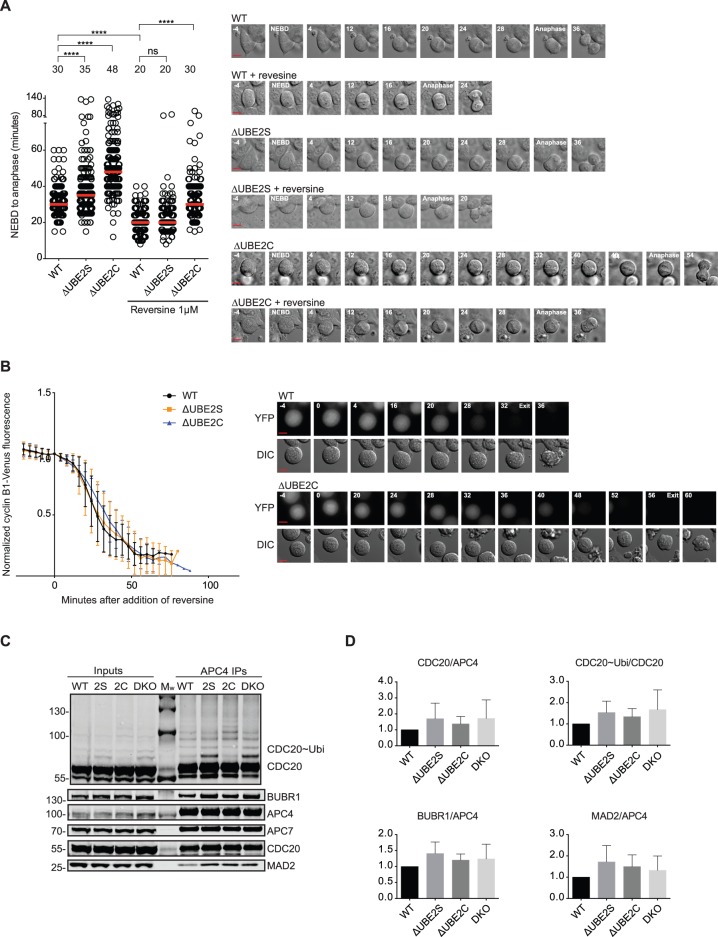
**Analysis of SAC silencing in cells lacking UBE2C or UBE2S.** (A) The indicated HCT116 cell lines were filmed either in the absence or presence of 1 μM reversine and the time from nuclear envelope breakdown (NEBD) to anaphase was determined. Each circle represents a single cell analyzed and the red line indicates the median, which is also stated in minutes above each condition. For each cell line, at least 154 cells were analyzed from three independent experiments. Representative stills on the right. ns, non-significant; ****P≤0.0001 by Mann–Whitney test. Scale bar in stills: 10 μm. (B) The indicated cells were arrested with nocodazole and then reversine was added and cyclin B1 degradation was determined by measuring the decrease in fluorescence signal in single cells. At least 20 cells from two independent experiments were analyzed for each cell line and the mean±standard deviation (s.d.) is indicated. Scale bar in stills: 10 μm. (C) The APC/C was immunopurified using an anti-APC4 antibody from the indicated cell lines treated with nocodazole and harvested by mitotic shake-off. The samples were analyzed for the indicated proteins by quantitative western blot. 2S, ΔUBE2S; 2C, ΔUBE2C; DKO, ΔUBE2C/ΔUBE2S. (D) Protein levels were normalized to APC4 levels and parental cell line (WT) set to 1. In the case of Cdc20 ubiquitination the first four bands of ubiquitination was measured and normalized to Cdc20. Mean±standard deviation (s.d.) from three independent experiments. There are no statistical significant differences (*t*-test).

To monitor the contribution of the E2 enzymes in SAC silencing, we performed time-lapse analysis of the mitosis of the parental HCT116 cell line, the ΔUBE2C cell line and the ΔUBE2S cell line (Fig. 1A). The time from nuclear envelope breakdown (NEBD) to anaphase onset is determined by the time it takes to bi-orient all chromosomes, how efficient the APC/C is in ubiquitinating its substrates (APC/C activity) and how efficiently the SAC is silenced. The time to bi-orient chromosomes and SAC activity is similar in all cell lines (Wild et al., 2016) and so differences in NEBD-anaphase times largely reflect the activity of the APC/C and how efficiently the SAC is silenced at metaphase. We analyzed the mitotic duration in the absence and the presence of the Mps1 inhibitor reversine (Santaguida et al., 2010), which results in inactivation of the SAC, and thus provides a readout of differences in APC/C activity. The median time from NEBD-anaphase was 30 min in the parental cell line and 20 min in the presence of reversine, and similar results were observed for the ΔUBE2S cell line (median 35 min and 20 min in reversine; Fig. 1A). In the ΔUBE2C cell line, the NEBD-anaphase time was 48 min and 30 min in the presence of reversine. From this we conclude that the maximum contribution to SAC silencing in HCT116 cells during an unperturbed mitosis is 8 min from UBE2C (18 min difference in the absence of reversine of which 10 min can be contributed to the lower APC/C activity in ΔUBE2C cells) and 5 min from UBE2S (5 min difference in the absence of reversine but no difference in APC/C activity).

To further explore the contribution of the E2 enzymes in SAC silencing, we challenged the different cell lines with nocodazole to depolymerize the microtubules and thus mount a checkpoint response. To these cells, we added reversine to inhibit the kinetochore-derived SAC signal and then monitored how fast cells exited mitosis, providing a read-out of SAC silencing efficiency. The parental cells exited with a median time of 31 min, the ΔUBE2S cells exited in 40 min and the ΔUBE2C cells in 47 min (Fig. S1A). Assuming these differences are solely due to an effect on SAC silencing the contribution of UBE2S would be 9 min and that of UBE2C 16 min.

To more directly monitor the activation of APC/C-Cdc20, we used our E2 knockout cell lines in which the endogenous cyclin B1 was tagged with the Venus fluorescent protein (cyclin B1-Venus) and used the reduction in fluorescence as a read-out of timing of APC/C-Cdc20 activation and its activity (Clute and Pines, 1999). We arrested cells with nocodazole and then added reversine as above and quantified the fluorescent signal as cells exited (Fig. 1B). We could not detect any significant differences in the onset of cyclin B1 degradation nor in the rate of degradation in cells lacking either UBE2C or UBE2S in this assay. This suggests that upon addition of reversine the APC/C is activated with the same efficiency in the complete absence of either UBE2C or UBE2S in HCT116 cells. At present we do not know why the morphological-based assays (Fig. 1A; Fig. S1A) reveal differences between the different cell lines while the cyclin B1 degradation assay does not.

As checkpoint silencing mechanisms are constantly antagonizing the kinetochore-derived checkpoint signal (Varetti et al., 2011), a weakening of silencing mechanisms should result in increased levels of MCC components on the APC/C as observed in cells depleted of p31^comet^ (Westhorpe et al., 2011). To investigate this, we immunopurified the APC/C complex from nocodazole-arrested cells and monitored MCC binding (Fig. 1C,D). We did observe an increase in MCC components in the E2 knockout cells but none of the increases were statistically significant (*t*-test, Fig. 1C,D; see also Fig. 4 using a different synchronization and purification protocol that revealed an increase in ΔUBE2C cells). The removal of UBE2S did result in more mono-ubiquitinated Cdc20 as expected but the total level of Cdc20 ubiquitination did not change when we quantified the first four bands of ubiquitinated Cdc20 species (the ubiquitinated species of Cdc20 that could be robustly quantified). In these experiments, we included a double knockout E2 cell line (ΔUBE2CΔUBE2S, referred to as DKO in figure) and the MCC levels in APC/C immunopurifications were also increased in this cell line but again this was not statistically significant.

In conclusion, these experiments show a minor contribution of the E2 module in SAC silencing *in vivo* in HCT116 cells.

### A synergistic effect on mitotic progression by removing APC15 in cells lacking UBE2C

We speculated that the function of the E2 enzymes potentially was acting in concert with other SAC silencing components such as p31^comet^ or APC15 and that in the absence of the E2 enzymes cells might become more sensitized to removal of these. Indeed, UBE2C has been proposed to act in concert with p31^comet^ in SAC silencing by promoting Cdc20 ubiquitination (Reddy et al., 2007).

**Fig. 2.**
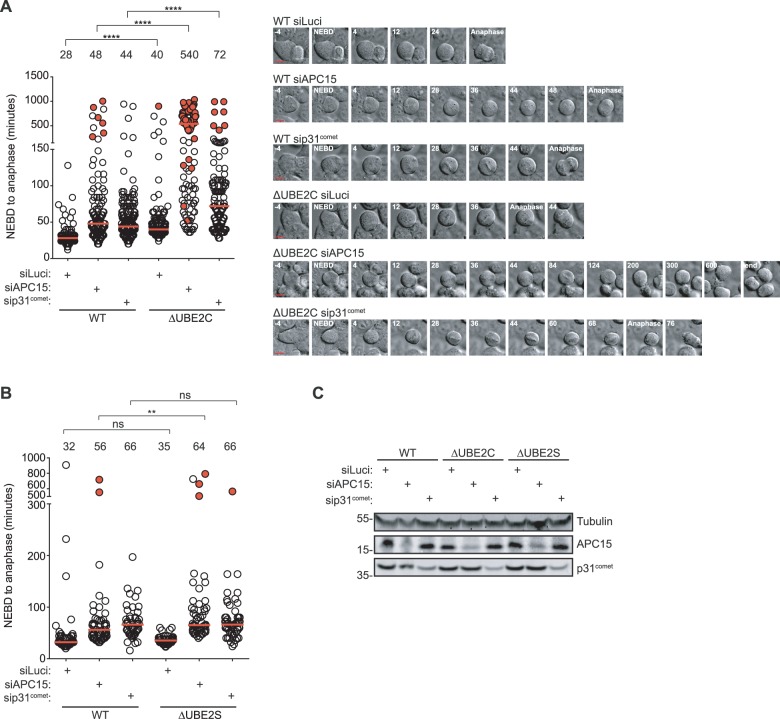
**APC15 depletion induces a strong mitotic arrest in ΔUBE2C cells.** (A,B) The indicated cell lines were treated with siRNAs targeting luciferase as a control, APC15 or p31^comet^ and filmed as they progressed through mitosis. Each circle represents a single cell analyzed and the red line indicates the median, which is also stated in minutes above each condition. Red circles represents cells that were still arrested when recording ended. Representative stills on the right. Scale bar in stills: 10 μm. ns, non-significant; **P≤0.01, ****P≤0.0001 by Mann–Whitney test. For A, at least 121 cells per condition were analyzed from two independent experiments. (B) Representative experiment with at least 48 cells analyzed per condition. (C) Western blot analysis of lysates from the indicated cell lines showing the levels of APC15 and p31^comet^. The depletion efficiencies in the different conditions were: WT, 55% p31 and 51% APC15; ΔUBE2C, 64% p31 and 44% APC15; ΔUBE2S, 63% p31 and 57% APC15 when normalized to luciferase and setting control depletion to one.

To explore this, we depleted p31^comet^ and APC15 by approximately 60% and 50%, respectively, using RNAi in the different cell lines and monitored mitotic progression (Fig. 2A-C, see figure legend for depletion efficiencies). Strikingly, the depletion of APC15 specifically in cells lacking UBE2C resulted in a pronounced synergistic effect on mitotic timing. This was in contrast to the depletion of p31^comet^ in ΔUBE2C or the depletion of APC15 in ΔUBE2S that resulted in an almost additive effect. At present, we do not want to exclude that a more penetrant p31^comet^ depletion could induce a pronounced delay in ΔUBE2C cells, but we note that the effect of APC15 depletion and p31^comet^ depletion results in almost similar mitotic delays in the parental cell line. The mitotic delay induced by our APC15 RNAi could be rescued by an RNAi resistant version of APC15 in HeLa cells and a synergistic inhibition of mitotic progression in the ΔUBE2C cell line was obtained with other distinct APC15 RNAi oligoes (Fig. S1B,C).

**Fig. 3.**
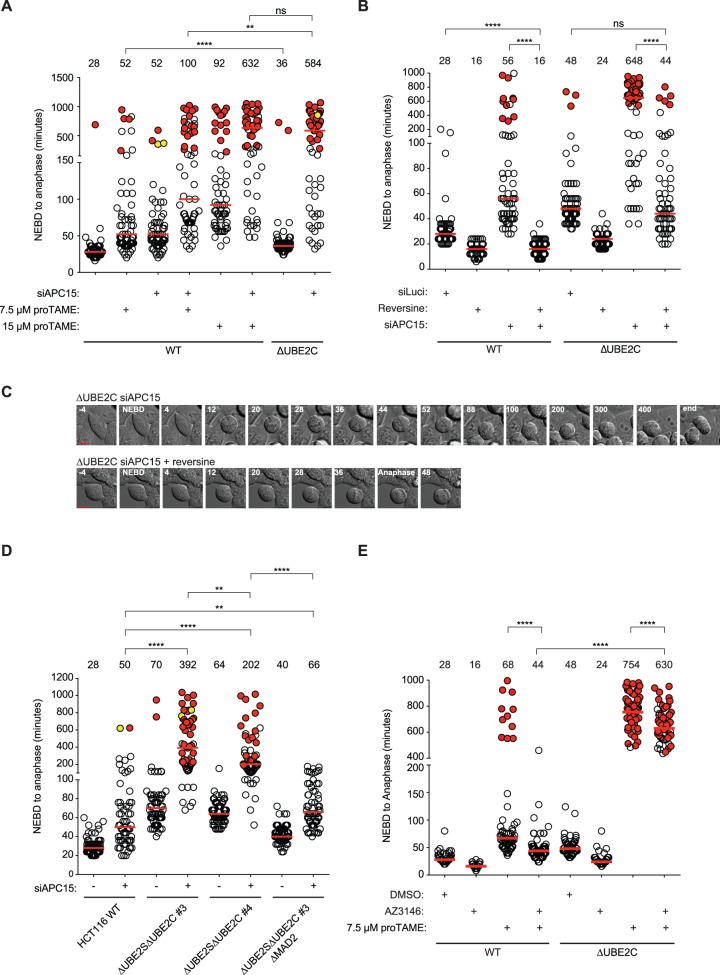
**The effect of APC15 but not pro-TAME is checkpoint dependent.** (A,B) The indicated cell lines were depleted of APC15 or treated with the indicated concentrations of pro-TAME or reversine and mitotic timing was analyzed by time-lapse microscopy. Each circle represents a single cell analyzed and the red line indicates the median, which is also stated in minutes above each condition. Red circles represent cells that did not exit mitosis within the stated time. Yellow circles represent cells that died at the indicated times. ns, non-significant; **P≤0.01, ****P≤0.0001 by Mann–Whitney test. For A, at least 59 cells were analyzed from two independent experiments. For B, at least 60 cells were analyzed from two independent experiments. (C) Representative stills for B. Scale bar: 10 μm. (D) APC15 was depleted in the indicated cell lines and mitotic timing analyzed by time-lapse microscopy. Red-filled circles represent cells that did not exit mitosis within the stated time. Yellow-filled circles represent cells that died at the indicated times. At least 60 cells were analyzed from two independent experiments. **P≤0.01, ****P≤0.0001 by Mann–Whitney test. (E) Indicated cell lines were treated with 7.5 μM pro-TAME and then either DMSO or the Mps1 inhibitor AZ3146 as indicated and mitotic timing analyzed by time-lapse microscopy. At least 60 cells were analyzed from two independent experiments. ****P≤0.0001 by Mann–Whitey test.

To explore if reduction of APC/C activity per se resulted in synergy with APC15 depletion, we treated cells with the APC/C inhibitor pro-TAME at 7.5 μM and 15 μM (Fig. 3A). Depleting APC15 by RNAi in cells treated with pro-TAME resulted in a synergistic inhibition of mitotic progression, particularly evident at the highest concentration of pro-TAME, showing a general sensitization of the APC/C complex to further inhibition upon depleting APC15 (Fig. 3A). The effect of APC15 RNAi was dependent on an active checkpoint as the effects could be almost fully reversed by Mps1 inhibition (Fig. 3B,C; Fig. S1E). Indeed, APC15 RNAi in cells lacking both UBE2C and UBE2S also resulted in a strong mitotic arrest that was dependent on Mad2 (Fig. 3D). This was in contrast to the effect of pro-TAME on ΔUBE2C cells or ΔUBE2SΔUBE2C that was almost checkpoint independent (Fig. 3E; Fig. S1D). We note that depletion of APC15 in ΔUBE2SΔUBE2CΔMAD2 cells (Fig. 3D) or in ΔUBE2C cells treated with reversine (Fig. 3B) resulted in an increased time of mitotic progression suggesting that the removal of APC15 might slightly perturb APC/C activity independently of the SAC – an effect that is only observed under conditions were APC/C activity is reduced such as through UBE2C removal.

**Fig. 4.**
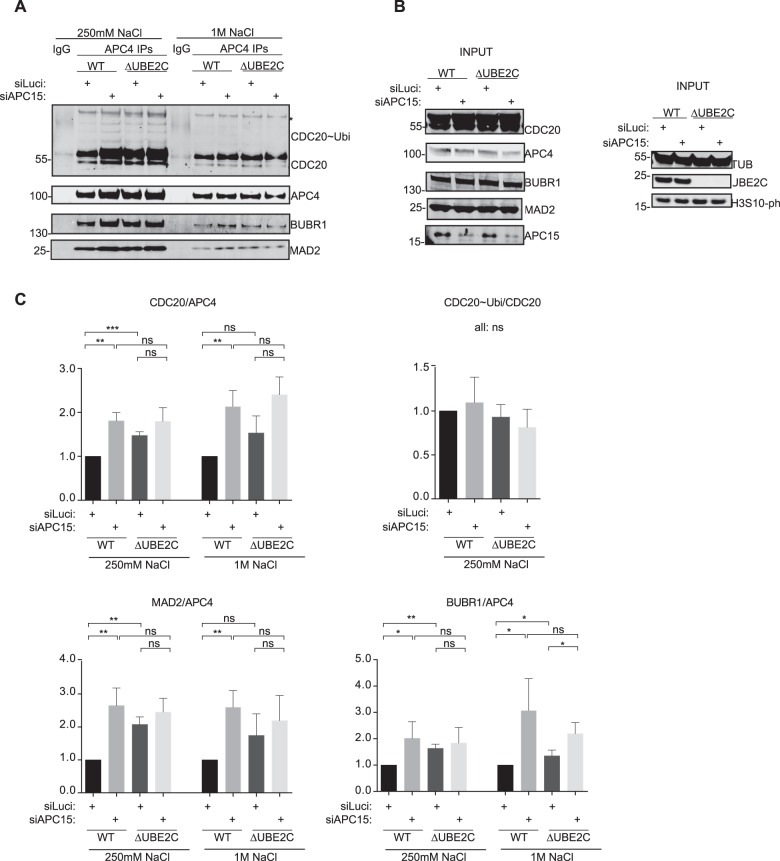
**APC/C-MCC interaction in ΔUBE2C cells depleted of APC15.** (A) The APC/C was purified in the presence of 250 mM NaCl or 1 M NaCl using an anti-APC4 antibody and purifications analyzed for MCC components by western blot. The asterisk indicates an unspecific band in Cdc20 blot. (B) Input for purifications in A. (C) Quantification of the levels of Cdc20, Mad2 and BubR1 normalized to APC4 and WT set to 1. Cdc20 ubiquitinated species are normalized to Cdc20. The mean±standard deviation (s.d.) is indicated. ns, non-significant; *P≤0.1; **P≤0.01; ***P ≤0.001 by *t*-test.

Our results so far argue for a strong synergistic effect between the removal of UBE2C and APC15 – an effect that is checkpoint-dependent. As APC15 depletion in HeLa cells results in increased levels of MCC on the APC/C (Mansfeld et al., 2011; Uzunova et al., 2012), we explored if the strong effect of APC15 RNAi in ΔUBE2C cells was a result of strongly increased levels of MCC on the APC/C in this specific situation. We immunopurified the APC/C and measured changes in MCC levels upon APC15 depletion (Fig. 4). To reveal potential differences in the strength of interaction between the APC/C and the MCC complex, the purifications were washed with buffer containing either 250 mM or 1 M salt. We did observe an increase in MCC components on the APC/C upon depletion of APC15 in wild-type HCT116 cells but this was not increased further when APC15 was depleted in ΔUBE2C cells. The stability of the APC/C-MCC interaction was also similarly affected by increased salt in the different samples. This suggests that the effect on mitotic progression by APC15 depletion in cells lacking UBE2C cannot be explained by increased levels of the MCC on the APC/C or an increase in the salt stability of the complex.

## DISCUSSION

The timely and rapid activation of the APC/C is critical for chromosome segregation and here we have explored the role of the APC/C-associated E2 enzymes, UBE2C and UBE2S, in SAC silencing. Using genetically engineered HCT116 cells null for either UBE2C or UBE2S, we have analyzed SAC silencing in both unperturbed and forced SAC inactivation assays. We detect a small contribution of the E2 enzymes in SAC silencing in these assays, but clearly the SAC can still be silenced efficiently in HCT116 cells lacking either UBE2C or UBE2S. Prior works have argued for a contribution by the E2 enzymes in SAC silencing in other cell lines (Garnett et al., 2009; Reddy et al., 2007). It is possible that SAC silencing in the cell lines used in these studies are more dependent on the E2 enzymes but we note that at least one study using a forced SAC inactivation assay could not detect any effect of depleting UBE2S in HeLa cells (Fig. 5 in Garnett et al., 2009).

The most pronounced effect was observed when cells lacking UBE2C were depleted of APC15, which resulted in a strong synergistic inhibition of mitotic progression. Our biochemical analysis of the APC/C-MCC interaction did not identify the molecular mechanisms behind this synergy but our results do not support increased levels of the MCC on the APC/C as the cause. The recently elucidated structures of APC/C-MCC complexes reveal that the MCC can block UBE2C binding when the MCC is in a closed conformation and that removal of APC15 results in a higher proportion of MCC in this closed conformation (Alfieri et al., 2016; Yamaguchi et al., 2016). One possible explanation for the observed synergy between APC15 depletion and the lack of UBE2C is that APC15 removal, through the MCC, interferes with the binding of the remaining initiating E2 enzyme UBE2D. This would be in agreement with our observation that modest depletion of UBE2D synergizes with the absence of UBE2C in causing a mitotic delay (Wild et al., 2016).

The results presented here provide *in vivo* insight into the mechanisms regulating the APC/C and pinpoint effective mechanisms to inhibit this complex for potential future therapeutic uses.

## MATERIALS AND METHODS

### Cell culture

HCT116 cells, HeLa-FRT and HeLa FLAG-APC15 cells were maintained in DMEM supplemented with 10% fetal bovine serum (FBS, HyClone) and 1% penicillin-streptomycin (Life Technologies). HeLa FLAG-APC15 cells media were supplemented with 200 mg/ml Hygromycin B (Invitrogen) and 700 ug/ml G418 (Gibco). Protein expression was induced by treatment with 1 ng/ml doxycycline (Clontech Laboratories).

### Microscopy

Cells grown in an 8-well chamber (Ibidi) with L-15 medium (Gibco) supplemented with 10% FBS were mounted on a DeltaVision Elite microscope (GE Healthcare) using a 40× oil-immersion objective (1.35 NA, WD 0.10). The images (DIC or YFP) were acquired with 5 or 4 min intervals for 4-18 h, taking three *z*-stacks of 7 μm. SoftWork software (GE Healthcare) was used for data analysis. ImageJ (NIH) was used to extract still images.

### Drug treatments

AZ3146 (2 µM, Tocris) and proTAME (Boston Chemicals) were added just prior to filming at indicated concentrations. Cells were treated with 25 ng/ml nocodazole (Sigma Aldrich) overnight to arrest them. For SAC silencing assays (Fig. S1A) 12.5 ng/ml nocodazole was used in the media and reversine (Cayman Chemicals) added after 60 min of filming. Cyclin B1 degradation assays were performed similarly but with 25 ng/ml nocodazole.

### RNAi mediated knockdown

The seeded cells were transfected with siRNAs (with final concentration of 50 nM or 10 nM for silencer select) in Opti-MEM medium (Gibco) for 6 h. The medium was changed to DMEM supplemented with 10% FBS. Double knock down was performed for all experiments.

All siRNA oligoes used were obtained from Sigma. As a negative control, siLUCI was used: 5′-CGUACGCGGAAUACUUCGA-3′. The following siRNA oligo was used for APC15 depletion: 5′-GUCUGGUCUAAGUUUCUUU-3′. Three different silencer select oligoes were used when indicated for APC15 depletion (s24721, s24722, s24723). For p31^comet^ depletion the following siRNA oligo was used: 5′-GGCUGCUGUCAGUUUACUUtt-3′.

### Immunoprecipitation

To synchronize cells for immunoprecipitation, a double (Fig. 1C,D) or single (Fig. 4A-C) thymidine (2.5 mM, Sigma) block overnight was followed by 6 h of release and then 200 ng/ml nocodazole was added. Cells were collected by mitotic shake-off. Cells were lysed in 150 mM NaCl lysis buffer and the lysates were incubated with APC4 or IgG Sepharose 4B cross-linked beads. Three 10 min washes with 250 mM NaCl lysis buffer were done before LDS loading buffer elution.

For MCC stability assays, the incubation of lysates with cross-linked beads was followed by one 10 min wash with low salt lysis buffer and afterwards incubated for 30 min with buffer containing either 250 mM or 1 M NaCl.

### Antibodies

The following antibodies were used for immunoprecipitation and western blotting as indicated: APC4 (1:500; mouse, prepared in-house), CDC20-p55 (1:100; mouse, E-7, Santa Cruz Biotechnology #sc-5296), APC7 (1:2000; rabbit, Bethyl #A302-551A-1), MAD2 (1:1000; rabbit, Bethyl #A300-301A), BUBR1 (1:500; rabbit, prepared in-house), APC15 (1:500; rabbit, gift from Jon Pines, Gurdon Institute, Cambridge, United Kingdom), APC15 (1:100; mouse, Santa Cruz #sc-398488), p31^comet^ (1:500; mouse, prepared in-house), UBE2S (1:500; rabbit, Abnova #PAB1701), UBE2C (1:500; rabbit, Boston Biochemicals #A-650), Tubulin (1:10,000; rat, Abcam #ab6160), and anti-phospho-histone H3 (Ser10) (1:1000; rabbit, Milipore #06-570).
